# Vibration prediction and analysis of the main beam of the TBM based on a multiple linear regression model

**DOI:** 10.1038/s41598-024-53868-6

**Published:** 2024-02-12

**Authors:** Yalei Yang, Lijie Du, Qingwei Li, Xiangbo Zhao, Zhihua Ni

**Affiliations:** 1 School of Mechanical Engineering, Shijiazhuang Tiedao University, Shijiazhuang, 050043 China; 2 Collaborative Innovation Center for Performance and Safety of Large-Scale Infrastructure, Shijiazhuang Tiedao University, Shijiazhuang, 050043 China; 3 Xinjiang Irtysh River Investment and Development (Group) Co., Ltd., Urumqi, 830000 China

**Keywords:** TBM, Multiple linear regression, Field penetration index, Driving power index, Vibration real-time prediction model, Engineering, Civil engineering, Mechanical engineering

## Abstract

The vibration of tunnel boring machine (TBM) is very difficult to monitor on sites, and related research on prediction methods is rare. Based on the field tunnelling test of a TBM in the Xinjiang Ehe project, the vibration information of the main beam of the TBM under different surrounding rock conditions is collected. The relationships among the tunnelling parameters, surrounding rock parameters and vibration parameters were studied. The results show that the penetration, cutter head speed, torque and thrust are important parameters affecting TBM vibration. In addition, the field penetration index and cutter head driving power index are significantly related to the root mean square of acceleration. Based on this, a multiple regression prediction model of TBM vibration is established. The model was verified and analysed via field projects, and the relative prediction error was less than 12%. This method can be used to predict the vibration of a TBM in real time through characteristic parameters without the use of a traditional monitoring system. This approach is highly important for determining the status of TBM equipment in real time.

## Introduction

Full-face tunnel boring machine (TBM) play an increasingly important role in the development and construction of underground spaces due to their safety and high efficiency^1,2^. In complex geological environments, a TBM is subjected to strong impact loads during the process of rock breaking, which causes severe vibrations of the main engine system of the TBM^3,4^. As the main engine system is a key component of a TBM, long-term severe vibrations can easily lead to serious engineering problems, such as bolt fracture and weld cracking, which directly affect the progress of construction projects. Furthermore, harsh working conditions create many difficult problems for the installation, protection, and data transmission of sensors. These issues make it very difficult to monitor TBM vibrations on-site. Thus, accurate prediction of TBM vibrations has become an important task in TBM tunnel engineering.

In recent years, scholars have made various achievements in terms of theoretical analyses and experimental tests on TBM vibrations. Using theoretical analysis, Sun et al.^5^ established a multi-degree-of-freedom coupled dynamic model of a TBM system. The cutter head, main beam, shield, and main bearing were all included in the model. It was concluded that the vibrations of the cutter head were primarily caused by external excitation. Based on the time-varying impact load from the TBM cutter head to the support boots, Huo et al.^6^ obtained the vibration acceleration range of the TBM cutter head in three directions by establishing a coupled nonlinear dynamics model. Zou et al.^7^ studied the coupling between the cutter head and main beam in the vibration direction by establishing a dynamic model of the rigid–flexible coupling of a TBM.

Using experimental testing, Zhang et al.^8^ collected data on the vibrations of a TBM cutter head based on the Yinsong project in Jilin, China. The results show that with an increase in penetration (P), the vibrations of the cutter head also strengthened. Based on engineering field tests, Yang et al.^9^ collected vibration signals from different types of surrounding rock and different tunnelling parameters using a vibration monitoring system. The vibration characteristics of the main beam of the TBM were revealed. Huang et al.^10^ developed a wireless sensor monitoring system for TBM cutter-head vibrations. The vibration signals of the cutter head were collected, which confirmed that the vibrations of the cutter head were related to the TBM tunnelling parameters and geological conditions. Zhou et al.^11^ relied on a water supply project in Northwest China, collected the vibration signals of the cutter head, and established their relationship with surrounding rock parameters. Yang et al.^12^ used laboratory tests to collect vibration data under various geological conditions. The vibration characteristics of the cutter head in soft and hard mixed strata were analysed. With respect to vibration prediction, Lin^13^ studied the propagation law of high slope blasting vibration and obtained a vibration velocity prediction model considering the elevation effect through dimensionless analysis. Wang et al.^14^ established a prediction model for the vibration velocity of shallow subway tunnels by combining numerical calculations with field monitoring. The results show that the predicted values of the numerical calculation method are in good agreement with the field measurements. Wang et al.^15^ improved the traditional BP neural network model, established a random reconnection BP neural network, and realized the amplified intelligent prediction of peak acceleration of accumulation slopes. Based on the research described above, most scholars have studied the vibrations and other related characteristics of TBMs by establishing dynamic models and using vibration data collected by sensor monitoring systems. At present, vibration prediction relies mainly on the vibration caused by blasting construction. However, TBM vibration prediction methods have rarely been studied, and the relationships among the vibration characteristics, tunnelling parameters, and geological parameters are unclear. Vibration sensor monitoring systems are easily damaged and exhibit poor reliability under harsh working conditions. Thus, real-time online monitoring of TBM vibrations is difficult. Thus, large amounts of TBM vibrations, tunnelling parameters, and geological information were collected in this study under different working conditions through in situ tunnelling tests to establish a dataset based on the Ehe (EH) Project in Xinjiang, China. Based on the Class II surrounding rock, a correlation analysis of the tunnelling parameters was carried out after data preprocessing, and the main tunnelling parameters were screened. The root mean square (RMS), a characteristic of the vibrations, and a mapping relationship between the tunnelling parameters and the RMS were obtained. The optimal model was determined using a goodness-of-fit coefficient. Considering the comprehensive influences of geological factors and tunnelling parameters, the field penetration index (FPI) and cutter-head driving power index (DPI) were selected as characteristic parameters. The correlations between the characteristic parameters and RMS were also analysed. Based on the characteristic parameters, a multivariate regression prediction model for the main beam vibrations of the TBM was established. To improve the applicability of the model, the distributions of the vibration characteristics of different surrounding rock types were statistically analysed. The ranges of the RMS correction coefficients were determined. A vibration prediction model of the main TBM beam suitable for different surrounding rocks was established. The model was validated and analysed for different surrounding rock types and equipment conditions, and its prediction effect was good. The model can effectively overcome the difficulties of field vibration monitoring and obtain the vibrations of the main TBM beam in real time without requiring the installation of a vibration sensing test system. Thus, this study provides a new method for monitoring TBM vibrations.

## Data acquisition

### Project overview

The total length of Tunnel Section VII of the Xinjiang EH project was 38.25 km. The lithology was mainly tuff mixed with tuff breccia, tuff sandstone, and granodiorite. The total proportion of actual Class II and Class III surrounding rock was 82.81%, that of Class IV and Class V surrounding rock was 17.19%, and the distribution was irregular. The burial depth of the tunnel is 475–640 m. The bid section has a two-TBM single-headed tunnelling construction.

**Figure 1:**
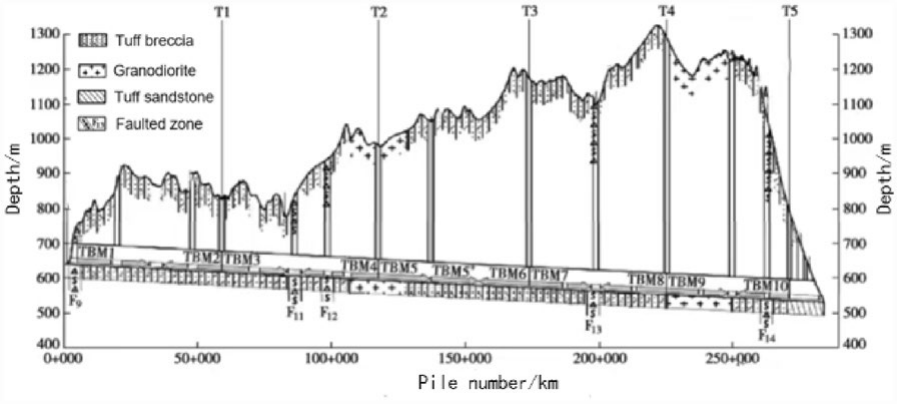
Geological profile of the tunnel in the Xinjiang EH project.

The two TBMs used were TBM7 and TBM8. They were assembled and excavated from the middle branch tunnel T4 using the "one hole and two machines" method. The tunnelling length of TBM7 was 17.88 km, whereas that of TBM8 was 19.64 km. A typical tunnel section of TBM7 was selected for conducting in situ excavation tests and establishing a prediction model. Typical tunnel sections of TBM7 and TBM8 were selected to verify and analyse the reliability and universality of the model. The geological profile of the tunnel in the Xinjiang EH project is shown in Fig. 1.

### Equipment parameters

The main technical parameters of the two open TBMs in Bid VII of the Xinjiang EH Project are listed in Table 1.

**Table 1:** Main technical parameters of the TBM.

Description	Parameter
Excavation diameter/mm	7030
Number of hobs	49
Cutter head driving power/kW	350 × 6 = 2100
Cutter head thrust/kN	23562@300 bar
Cutter head rotation speed/r min^−1^	0–10.6
Cutter head torque/kN m	4410@5.5 r/min
Cutter head relief torque/kN m	6620@0.5 r/min

### In situ tunnelling test

**Figure 2:**
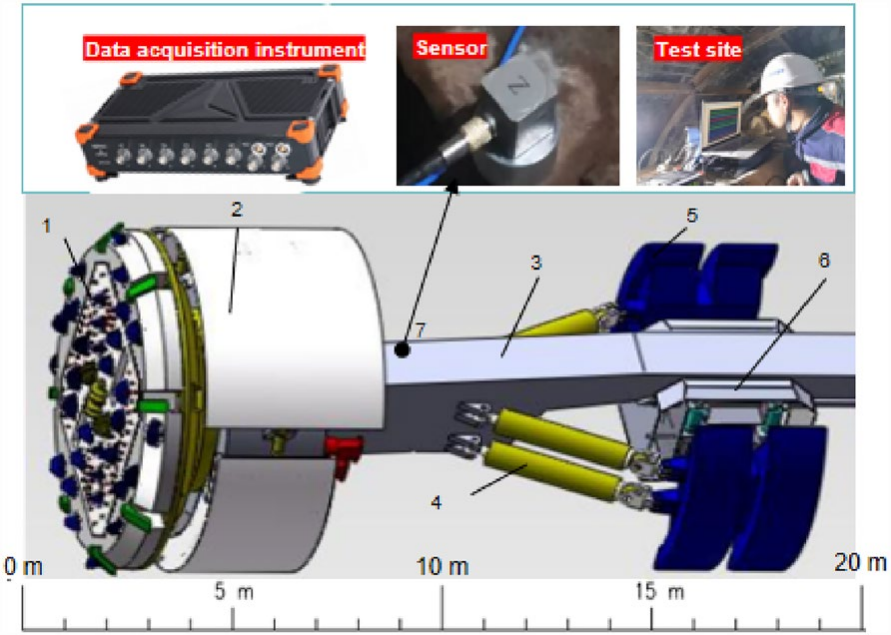
Schematic diagram of the TBM measuring point arrangement. 1- Cutterhead; 2- shield; 3- main beam; 4- propulsion cylinder; 5- support boots; 6- saddle frame; 7- measuring point.

Based on the Xianjiang EH project, a typical tunnel section was selected for in situ excavation tests. The tunnelling, geological, and vibration parameters of the main girder were acquired for the TBM under excavation. The tunnelling, geological, and main girder vibration parameters of the TBM under excavation were collected, and a database was established. The vibration measurement points were arranged in the front section of the main beam of the TBM, as shown in Fig. 2.

The main in situ tunnelling tests were as follows:

- Experiment 1: The cutter-head speed (N, r/min) was maintained constant, P was adjusted, and vibration information from the main beam of the TBM was collected.
- Experiment 2: P was maintained constant, N was adjusted, and vibration signals from the main beam of the TBM were collected.
- Experiment 3: When the TBM was operated under different surrounding rock conditions, the vibration information was collected throughout the entire process.

## Data preprocessing

The TBM data acquisition system recorded the entire tunnelling process, including non-tunnelling state data such as shutdown and step changes. Missing data or abnormal values may also be present. When the TBM was in the non-tunnelling state, the values of the main tunnelling parameters, including N, total propulsion force (F), cutter head torque (T), and P, were 0. On this basis, a binary decision function was set to filter out the non-tunnelling state data. Abnormal data were eliminated according to the Laida criterion^16–18^.

Because the main beam of a TBM must bear the alternating impact load caused by the cutter head breaking the rock, the vibration amplitude fluctuates widely. This increased the uncertainty when measuring the vibrations of the main beam. However, the RMS reflects the signal energy within a small range of fluctuations. The energy of the vibration signal of the main TBM beam is closely related to the surrounding rock conditions. The uncertainty caused by the impact load is eliminated. Therefore, it is reasonable to choose the RMS as the characteristic vibration quantity. In addition, because the vibrations along the tunnelling direction (i.e., the axial direction) are the largest, they contain the most useful information. The relationship between the axial RMS and tunnelling parameters was investigated in this study. The formula for calculating the RMS is as follows^19^:

1 $$RMS = \left( {\frac{1}{{\text{n}}}\sum\limits_{i = 0}^{n - 1} {x_{i}^{2} } } \right)^{\frac{1}{2}}$$

where x_i_ is the vibration acceleration at the sample point (m/s^2^).

## Data feature selection

**Figure 3:**
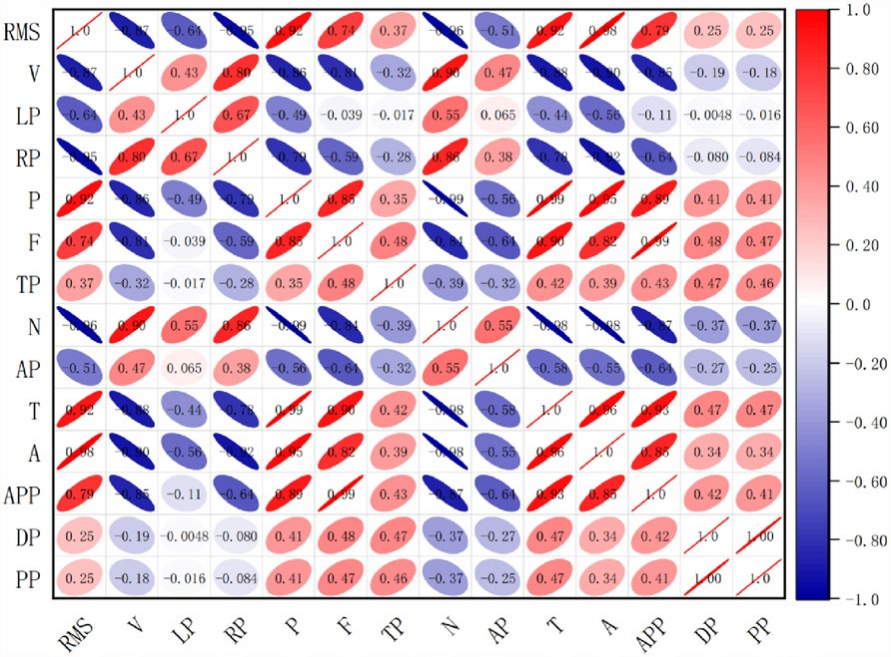
Correlation heatmap between the vibration characteristics and tunnelling parameters.

During TBM tunnelling, vibrations are influenced mainly by geological factors, equipment parameters, and tunnelling parameters. Based on the current geological conditions of the excavation and TBM equipment, the main influencing factors of the TBM vibrations are the tunnelling parameters. In situ tunnel tests were performed by selecting several typical tunnel sections. As many as 200 tunnelling parameters were collected during TBM tunnelling, and 30 groups of tunnelling parameters from the stable tunnelling sections and the RMS of the TBM vibrations were selected for Pearson correlation analysis. The correlation heatmap is shown in Fig. 3.

As shown in Fig. 3, P, F, N, T, the average current of the cutter head (A) and the advancing pump pressure (APP) were strongly related to the RMS of the main beam of the TBM. The correlations were 0.92, 0.74, -0.96, 0.92, 0.98 and 0.79, respectively. The correlations between P and F and between F and T are extremely strong. The reason is that the total F determines the interaction force between the disc cutter and the rock. The greater this force is, the greater the penetration depth of the disc cutter into the rock; that is, the greater the cutter head torque. The cutter head is driven by the motor, and F is realized by the power provided by the propulsion cylinder. Therefore, A and T have an inherent mutual feedback relationship, and F is a direct reflection of APP. Combined with the operation control mode of a TBM and the results of previous related research^20,21^ on the prediction of tunnelling parameters and the correlation between tunnelling parameters, we know that the total thrust, penetration, cutter head speed and cutter head torque are the four most important tunnelling parameters in the TBM tunnelling process. Thus, in this study, the correlations of P, F, N, and T with the RMS are focused on.

## Correlations between the vibration characteristics of the tunnel boring machine (TBM) main beam and the driving parameters

### Influence of the tunnelling parameters on the room mean square (RMS)

Because these are the main tunnelling parameters in the TBM tunnelling process, there is an inherent mutual feedback relationship between N, P, F, and T. In the process of tunnelling, TBM operators quickly adjust N and P based on the surrounding rock conditions and their previous experience. In general, when geological conditions are stable, N is determined based on the slag-tapping ability. However, due to changes in geological conditions, tool wear, and other conditions, P cannot be maintained at a fixed value but rather fluctuates around a set value. Then, F, the advancing speed (v, mm/min), and T were varied. The rock–machine interaction forces are further altered, which causes the TBM to vibrate^17^. Therefore, it is essential to study the correlation between tunnelling parameters and TBM vibrations to establish a rock–machine interaction model. In this study, the exponential, linear, polynomial, power function, and logarithmic models were used to regress the tunnelling parameters and RMS data. The coefficient of determination R^2^ was used to evaluate the performance of the regression models and establish the optimal regression model. R^2^ is defined as follows:

2 $$R^{2} = 1 - \frac{{\sum\limits_{i = 1}^{m} {(\tilde{y}_{i} - y_{i} )^{2} } }}{{\sum\limits_{i = 1}^{m} {(\overline{y}_{i} - y_{i} )^{2} } }}$$

where $\tilde{y}_{i}$ is the predicted sample value (m/s^2^), $\overline{y}_{i}$ is the mean value (m/s^2^), and $y_{i}$ is the actual value (m/s^2^).

#### Analysis of the influence of N on the RMS

**Figure 4:**
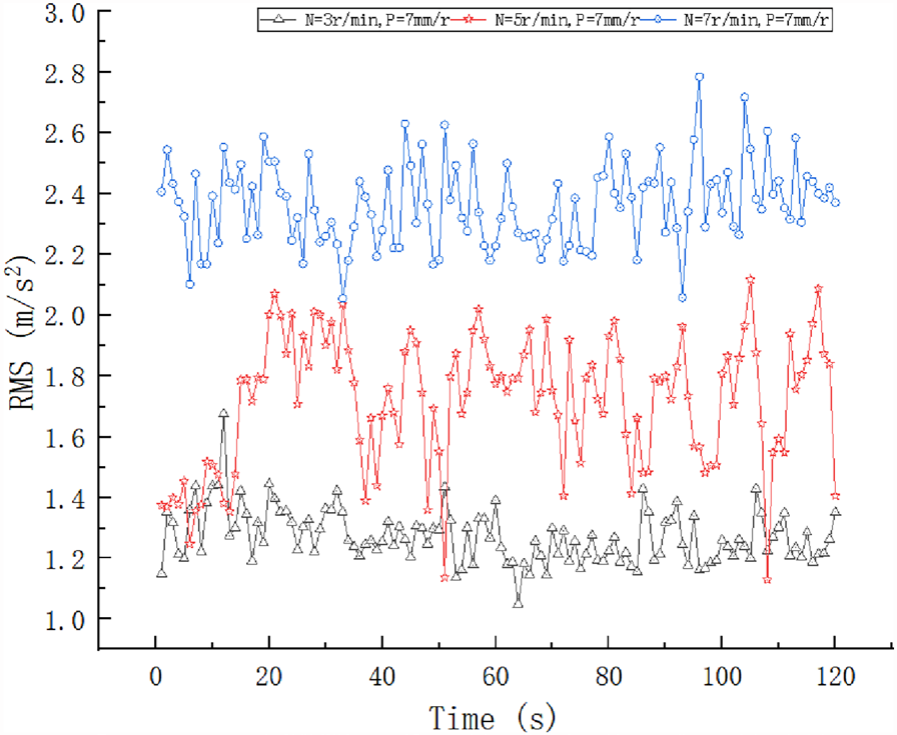
RMS results at different speeds.

The pile numbers of the test tunnel section ranged from 181 + 660.2 to 181 + 662.05, and the surrounding rock was Class II, which included tuffaceous sandstone and complete surrounding rock. The vibrations of the TBM at different cutterhead speeds (N = 3, 5, and 7 r/min) were monitored, and the penetration was fixed (P = 7 mm/r) to determine the influence of N on the RMS, as shown in Fig. 4.

As shown in Fig. 4, N had a significant influence on the RMS at the same P and different N values, and the RMS values were significantly different. As N increased, the RMS value also increased. When N was 7 rpm, the vibrations were the largest. The maximum RMS value was 2.79 m/s^2^, the minimum value was 2.06 m/s^2^, and the average value was 2.36 m/s^2^. The standard deviation was 0.13794 m/s^2^, and the fluctuation range was small. The second-largest vibration was observed for N = 5 r/min. The RMS average value was 1.72 m/s^2^, the standard deviation was 0.21569 m/s^2^, and the value fluctuated significantly. Because the geological conditions are complex and changeable, this may have been related to changes in the surrounding rock during data collection. When N was 3 r/min, the vibrations were the smallest, and the maximum and minimum RMS values were 1.6756 and 1.04902 m/s^2^, respectively. The average value was 1.2722 m/s^2^, and the standard deviation was the smallest at 0.08909 m/s^2^. The main reason was that N was low, the geology was uniform, and the impact was small. Therefore, the fluctuation range was the smallest.

#### Analysis of the influence of F on the RMS

**Figure 5:**
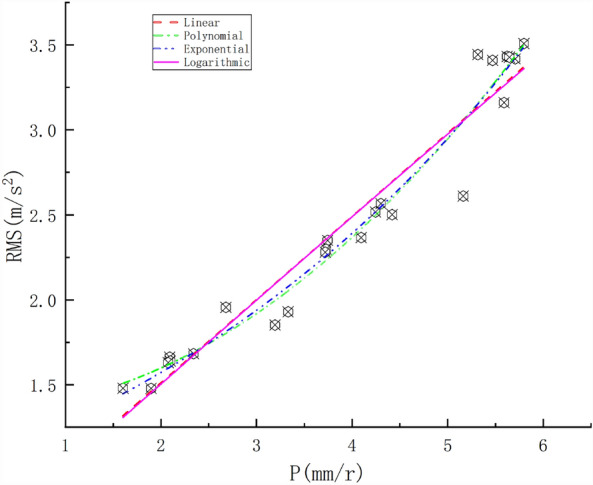
Correlations between penetration and RMS at different speeds.

The penetration was primarily affected by the total thrust and rock mass strength. The pile numbers of the test tunnel section were 158 + 145.44–158 + 153.78, and the surrounding rock was Class II with lithologic tuffaceous sandstone, an integrity coefficient > 0.75, a strength of 80–90 MPa, and N = 6.8 r/min. P was gradually increased, and the TBM vibration signal and tunnelling parameters were collected to establish a dataset. The relationship between P and the RMS was studied using a single-factor regression method. Empirical formulas were established by fitting the data with linear, polynomial, exponential, and logarithmic function models. The regression curves are shown in Fig. 5. The fitting formulas and R^2^ values for each regression model are listed in Table 2.

**Table 2:** Regression model of P and RMS.

Model	Fitting formula	R^2^
Linear	$RMS = 0.4896P + 0.52695$	0.93969
polynomial	$RMS = 0.0649P^{2} + 0.00583P + 1.34643$	0.95895
exponential	$RMS = 1.02967{\text{e}}^{0.21005P}$	0.96012
logarithmic	$RMS = 60.11689\ln (P + 119.13779) - 286.87171$	0.93544

Figure 5 shows that the RMS increased with an increasing P. The main reason is that when N is constant, the increase in P increases the driving speed, which causes the TBM to be subjected to a greater rock-breaking impact, leading to an increase in the TBM vibrations. This shows that penetration had a significant influence on the TBM vibrations. Regression analysis with linear, polynomial, exponential, and logarithmic functions yielded R^2^ values of 0.93969, 0.95895, 0.96012, and 0.93544, respectively. Thus, the exponential function had the best goodness-of-fit.

#### Correlations between F and the RMS

**Figure 6:**
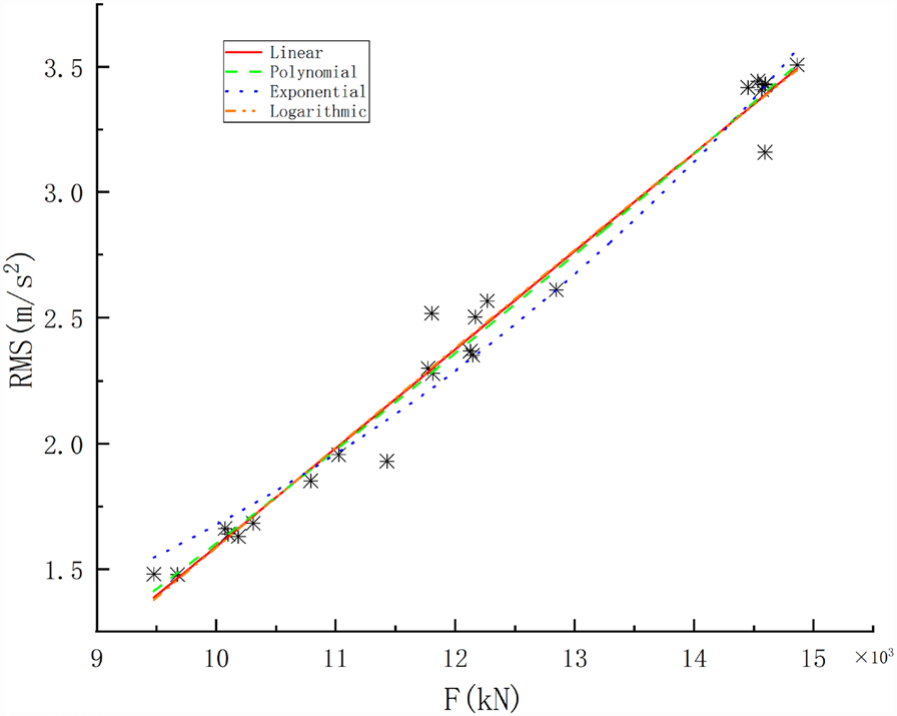
Correlations between the total thrust and RMS.

The total thrust is one of the most important parameters in TBM tunnelling. Based on the data collected for the test tunnel section piles with numbers 158 + 145.44–158 + 153.78, the relationship between F and the RMS was studied using the single-factor regression method. Empirical formulas were established by fitting and regressing the data with linear, polynomial, exponential, and logarithmic function models. The regression curves are shown in Fig. 6. The fitting formulas and R^2^ values for each regression model are listed in Table 3.

**Table 3:** Regression model of F and the RMS.

Model	Fitting formula	R^2^
Linear	$RMS = 3.91291 \times 10^{4} F - 2.3221$	0.98105
polynomial	$RMS = 5.16082 \times 10^{ - 9} F^{2} + 2.63705 \times 10^{ - 4} F - 1.55$	0.95895
exponential	$RMS = 0.35499e^{{1.55298 \times 10^{ - 4} F}}$	0.973
logarithmic	$RMS = 43.72224\ln (99430.59699 + F) - 505.72416$	0.97983

As shown in Fig. 6, the greater the F is, the greater the RMS vibration; that is, there is a positive correlation between them. In the process of rock breaking by a TBM, an increase in F directly leads to an increase in P. In an uneven geological environment, the TBM is subjected to fluctuating impact loads, and the RMS increases. The results showed that the influence of F on the RMS was significant. Regression analyses with linear, polynomial, exponential, and logarithmic functions were performed; as shown in Table 3, the R^2^ values were 0.98105, 0.98054, 0.973, and 0.97983, respectively. The R^2^ value of the linear function is the highest.

#### Correlations between T and the RMS

**Figure 7:**
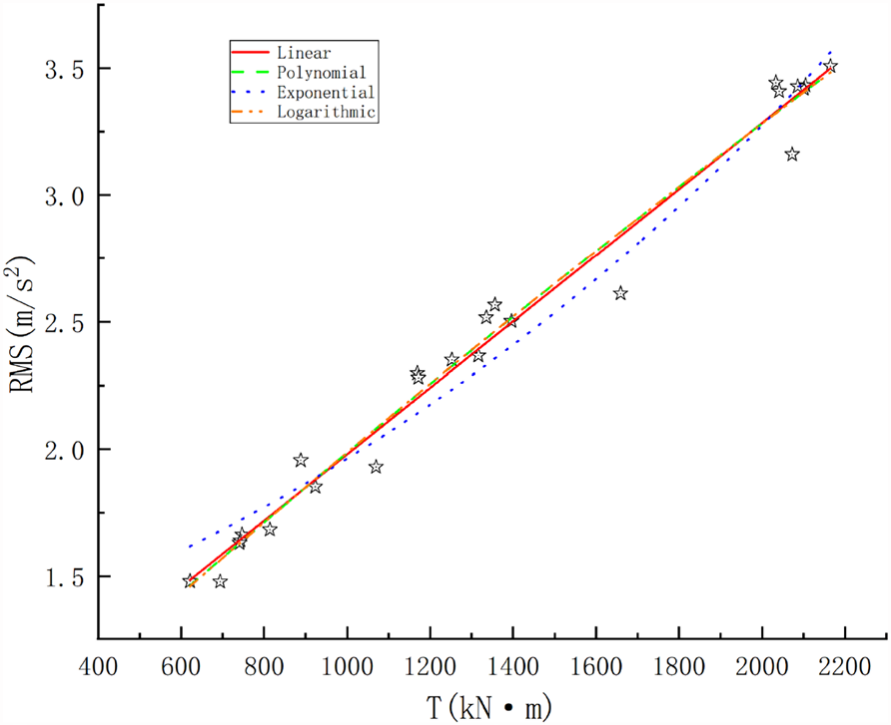
Correlations between the torque and RMS.

The torque drives the rotation of the cutterhead, which can be used to directly determine the value of N. Therefore, a change in this parameter will also cause a change in the vibrations. The data collected from the test tunnel with pile numbers 158 + 145.44–158 + 153.78 were used. The relationship between T and the RMS was also examined. Linear, polynomial, exponential, and logarithmic functions were used to fit the data and establish empirical formulas. The regression curves are shown in Fig. 7. The fitting formulas and R^2^ values for each regression model are listed in Table 4.

**Table 4:** Regression model of T and the RMS.

Model	Fitting formula	R^2^
Linear	$RMS = 0.0013T + 0.67542$	0.98127
polynomial	$RMS = - 6.25968 \times 10^{ - 8} T^{2} + 0.00148T + 0.00148$	0.98075
exponential	$RMS = 1.1768e^{{5.1169 \times 10^{ - 4} T}}$	0.96839
logarithmic	$RMS = 12.97405\ln (8529.70441 + T) - 116.88347$	0.98007

According to the analysis results in Fig. 7, the RMS was directly proportional to T; that is, with an increase in T, the RMS also increased. An increase in T corresponded to an increase in N, and the continuous acceleration of N caused disc cutter friction with the rock mass to produce a high temperature and bear a large impact load, leading to greater vibrations of the TBM. Linear, polynomial, exponential, and logarithmic fitting analyses of T and the RMS showed that there is a high correlation between these quantities. The corresponding R^2^ values are 0.98127, 0.98075, 0.96839, and 0.98007, respectively. The linear function exhibited the best fitting correlation. The results showed that T had a significant influence on the RMS.

### Tunnelling characteristic parameters

By analysing the correlations between key tunnelling parameters, such as F, P, N, and T, and the RMS, it was found that the four tunnelling parameters had a significant influence on the RMS under certain geological conditions. Furthermore, the tunnelling parameters were not completely independent of each other, and N and F were the main control parameters. The other tunnelling parameters were driven by and closely related to these parameters. The comprehensive influence of these four parameters should be considered when establishing the model.

#### Proposing characteristic parameters

There was a strong correlation between F and P of the TBM during tunnelling, and the single-cutter thrust required for each turn of the cutting depth (FPI) established a good relationship between them. The FPI is a good indicator of geological drivability. The larger the FPI is, the more difficult it is to drive, and vice versa. Thus, to comprehensively consider the influence of F and P on the RMS, the FPI can be used as a geological characteristic parameter to study correlations with the TBM vibration RMS. The formula for calculating the FPI is as follows:

3 $$FPI = F_{{\text{n}}} /P$$

where F_n_ is the single-cutter thrust (kN) and P is the penetration (mm/r).

The relationship between T and N can be established in terms of power to consider the comprehensive influences of N and T on the RMS. The cutter head driving power index (DPI) is proposed for this purpose and is defined as follows:

4 $$DPI = T \cdot N{/9550}$$

where T is the cutter head torque (kN m) and N is the cutter head speed (r/min).

#### Correlations between the characteristic parameters and RMS

**Figure 8:**
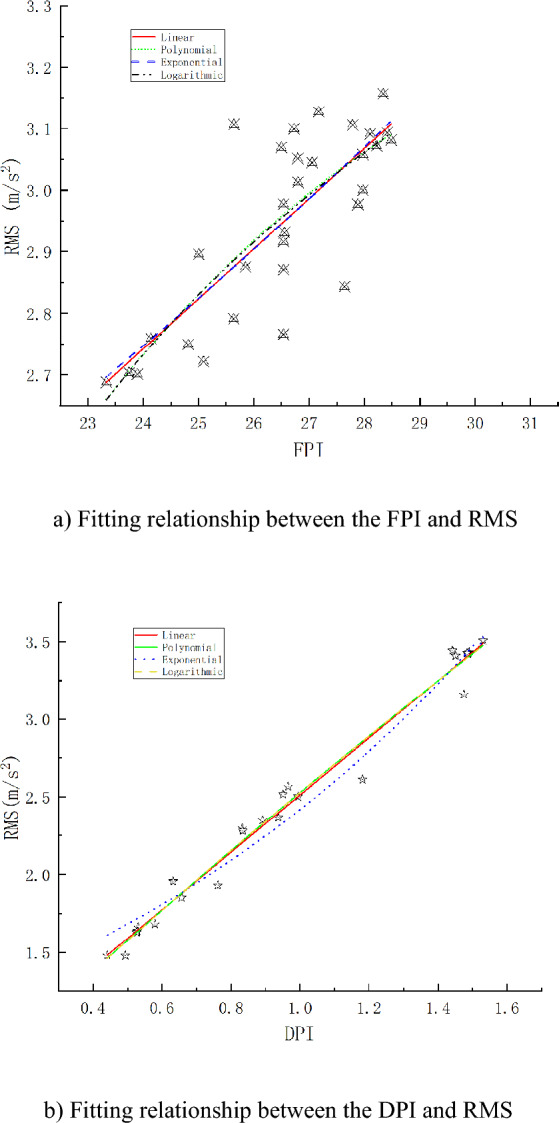
Correlations between characteristic parameters and vibration characteristic RMS.

The correlation between the characteristic parameters and the vibration RMS was analysed using the data collected for test tunnel section piles with numbers ranging from 158 + 145.44 to 158 + 153.78. The relationship between T and the RMS was also studied. Linear, polynomial, exponential, and logarithmic function models were used to fit the data and establish the empirical formulas. The regression curves are shown in Fig. 8. The fitting formula and R^2^ values of each regression model are listed in Tables 5 and 6, respectively.

**Table 5:** Regression model of the FPI and RMS.

Model	Fitting formula	R^2^
Linear	$RMS = 0.08145DPI + 0.78729$	0.62955
polynomial	$RMS = - 0.00532FPI^{2} + 0.35921FPI - 2.82479$	0.62281
exponential	$RMS = {\text{e}}^{0.02783FPI + 0.34197}$	0.62659
logarithmic	$RMS = 0.71462{\text{ln}}(FPI - 17.15164) + 1.35776$	0.62216

**Table 6:** Regression model of the DPI and RMS.

Model	Fitting formula	R^2^
Linear	$RMS = 0.084171DPI + 0.66902$	0.98094
polynomial	$RMS = - 0.10562DPI^{2} + 2.05638DPI + 0.57526$	0.98031
exponential	$RMS = 1.17249e^{0.72361 \cdot DPI}$	0.96859
logarithmic	$RMS = 15.29124\ln (DPI + 7.2955) - 29.8251$	0.98032

As shown in Fig. 8, the FPI and DPI were positively correlated with the RMS vibration; that is, with increasing FPI and DPI, the RMS vibration also increased. The FPI represents the difficulty in TBM tunnelling. The greater the FPI is, the greater the F required for tunnelling, but the smaller the value of P. The TBM was subjected to greater external excitation, which caused it to generate greater vibrations. The influences of N and T were comprehensively considered in the DPI. The greater the DPI is, the greater the energy required for TBM tunnelling. The TBM was also significantly impacted, resulting in significant vibrations. The R^2^ values for the fits of the FPI and RMS data with linear, polynomial, exponential, and logarithmic functions were 0.62955, 0.62281, 0.62659, and 0.62216, respectively. The corresponding values for the DPI and RMS data are 0.98094, 0.98031, 0.96859, and 0.98006, respectively. The R^2^ values of the linear fit were the largest, indicating that the optimal fitting models were linear.

## Establishment of the multiple linear regression model

Based on the above research and analysis, the four main tunnelling parameters of the TBM influence the RMS. When establishing a model of rock–machine interactions, the effects of the four factors should be fully considered, and the proposed characteristic parameters should take these factors into account. Therefore, a prediction model of the main beam vibrations of a TBM was established with the characteristic parameters FPI and DPI to reduce the number of input features, reduce the redundancy of the model, and further improve the operational efficiency of the model. A dataset of the RMS and tunnelling characteristic parameters was established using in situ tunnelling test data. The data were then subjected to multivariate linear regression using the multivariate regression method, and the function relating the RMS to the FPI and DPI was obtained as follows:

5 $$RMS = - 8.51421 \times 10^{ - 4} FPI + 1.80941DPI + 0.74997$$

The R^2^ value of the regression model is 0.98. The results show that 98% of the RMSs could be explained by the characteristic parameters FPI and DPI, which were significantly correlated with the RMS.

**Figure 9:**
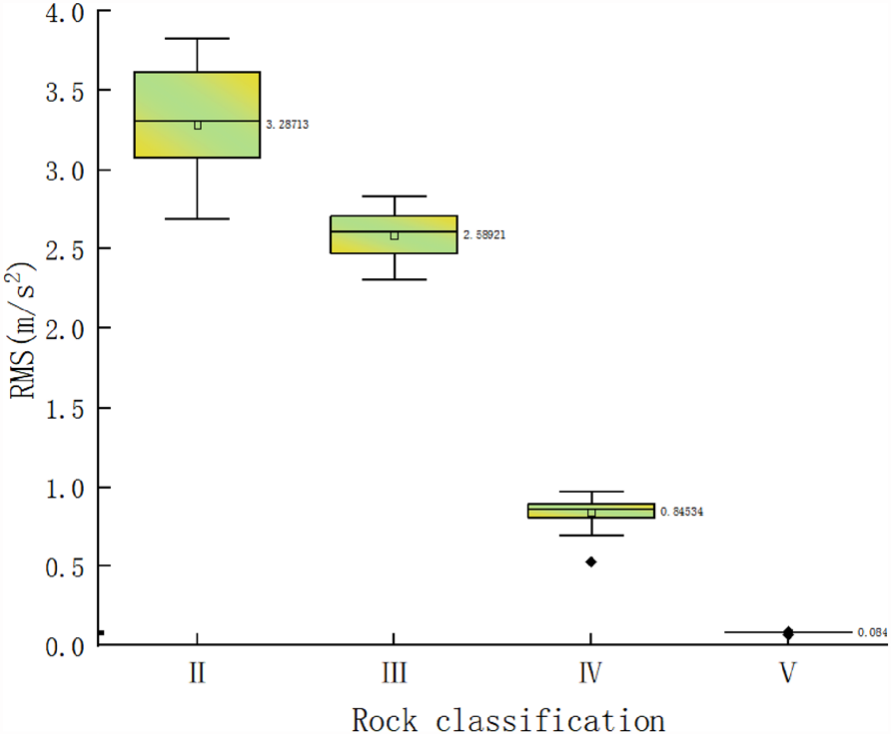
RMS distribution law for different surrounding rock types.

For Class II, III, IV, and V surrounding rocks, the vibration signals were collected continuously for 30 min at a sampling frequency of 1000 Hz and then averaged every 1 min. After the statistical analysis, the RMS distributions for the different surrounding rock types were obtained, as shown in Fig. 9. The results of the RMS statistical analysis are presented in Table 7.

**Table 7:** RMS statistical analysis of vibration under different surrounding rock types.

Class	Max	Min	Mean	IQR	Std
II	3.83	2.68766	3.28713	3.30978	0.35361
III	2.83536	2.30662	2.58921	2.61364	0.15109
IV	0.97333	0.52807	0.84534	0.86065	0.08192
V	0.08736	0.07052	0.08438	0.0849	0.00272

The statistical analysis results in Fig. 9 and Table 7 show that the RMS distribution ranges of the vibrations were different for the different surrounding rock types. Overall, as the number of surrounding rock types increased, the RMS decreased. The vibrations were the largest for the Class II surrounding rock. The average RMS was 3.29 m/s^2^. The vibrations were the smallest for Class V surrounding rock, and the mean RMS was 0.08438 m/s^2^. This result occurred mainly because the integrity and strength of the rock generally decreased with an increase in the number of surrounding rock types. As a result, the tunnelling parameters, such as the thrust required for TBM rock breaking, decreased. Thus, the TBM vibrations were reduced. Furthermore, the impact force on the TBM decreases when the surrounding rock deteriorates and the strength decreases. The RMS standard deviation (Std) was the largest for the Class II surrounding rock, with a value of 0.35361 m/s^2^, indicating that the vibration dispersion degree was the largest. The RMS Std of the Class V surrounding rock was the smallest, with a value of 0.00272 m/s^2^. In other words, the degree of dispersion was the smallest. The differences between the variation ranges of the Class II, III, and IV surrounding rocks and that of the Class V surrounding rocks were 21.23%, 67.43%, and 90.01%, respectively, which were relatively large. Therefore, the RMS was normalized between 0 and 1 as follows:

6 $$\beta_{{\text{i}}} = \frac{{X_{{\text{i}}} }}{{X_{\max } - X_{\max } }}$$

where X_i_ denotes the RMS and β_i_ denotes the normalized RMS.

A larger RMS indicates that the quality of the surrounding rock is better, and the surrounding rock is more stable. Based on the stability of the Class II surrounding rock, the vibration correction coefficient ($\alpha$) was set for the different types of surrounding rock. The normalized RMS was statistically analysed, and the vibration correction coefficient ranges for the different surrounding rock types were obtained using the median and mean values. The formula used is as follows:

7 $$\alpha_{{\text{i}}} = \frac{{1 - \beta_{{\text{i}}} }}{{1 - \beta_{2} }}$$

where β_2_ is the normalized value of the RMS for Class-II rock.

The RMS correction factors of the different surrounding rock types are shown in Table 8.

**Table 8:** RMS correction coefficient of different surrounding rock types.

Class	II	III	IV	V
*α*	1	0.92–0.98	0.32–0.37	0.03–0.04

By combining the RMS correction coefficients for different surrounding rock types, the RMS prediction model for the different surrounding rock types can be obtained as follows:

8 $$RMS = \alpha \cdot ( - 8.51421 \times 10^{ - 4} FPI + 1.80941DPI + 0.74997)$$

## Validation and analysis of the prediction model

**Figure 10:**
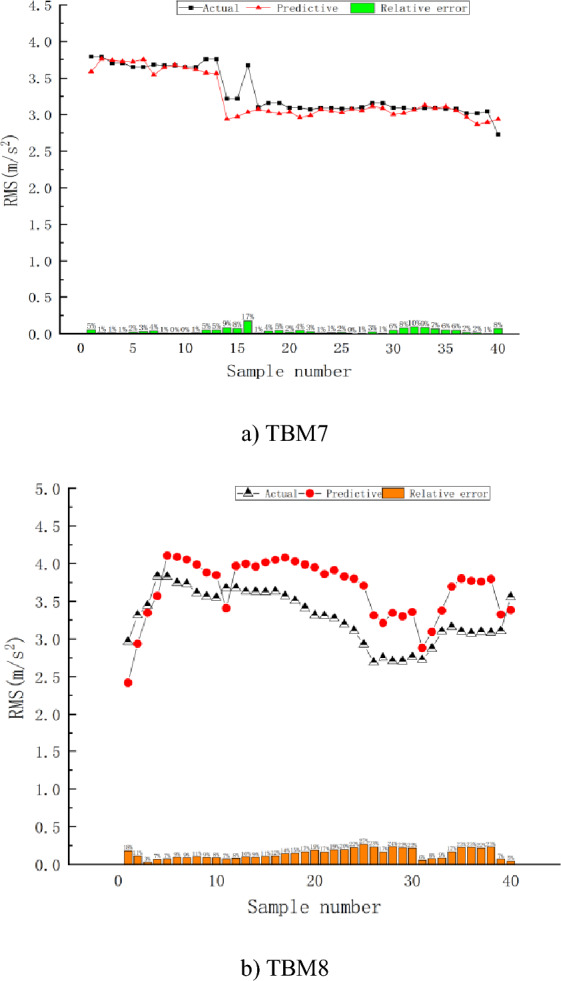
Comparison between the actual and predicted values.

To test the prediction effect of the established model, the TBM7 tunnel section piles 158 + 228.26–158 + 253.26 were selected. The surrounding rock was Class II, with tuffaceous sandstone, good rock integrity, and underdeveloped cracks. In situ excavation tests were conducted to test the accuracy of the prediction model, and the entire excavation process was normally driven by the main driver of the TBM. The collected vibration characteristic RMS was the average value, and the values of the characteristic parameters at each minute were collected as a set of data. Subsequently, the relative error between the actual and predicted values of the RMS was analysed by randomly selecting 40 groups of data from the stable driving section. Moreover, to verify the reliability of the model, a tunnelling test was carried out, and TBM8 tunnel section pile numbers 181 + 660.2–181 + 662.05 were used. The surrounding rock is Class II with good rock integrity, and the lithology is granodiorite. Subsequently, the data of 40 groups of stable tunnelling sections were randomly selected for comparative analysis. A comparison of the actual and predicted values of the RMS for the Class II surrounding rock is shown in Fig. 10. The results of the statistical analysis are listed in Table 9.

**Table 9:** Statistics of the RMS actual value and predicted value under Class II.

TBM	Actual value			Predicted value			Relative error (%)		
	Max	Min	Mean	Max	Min	Mean	Max	Min	Mean
TBM7	3.79	2.73	3.30	3.76	2.87	3.23	17.39	0.14	3.85
TBM8	3.83	2.69	3.29	4.11	2.42	3.66	26.61	2.76	14.23

A comparison and analysis of the predicted and actual values in Fig. 10 and Table 9 reveal that the predicted RMS values are very close to the actual values. The maximum relative error (RE) in the prediction was 17.39%. The average relative error was 3.85%. Thus, the prediction was good. The main reason for this was that the test conditions of the verification analysis and prediction model were relatively consistent. The TBM equipment was unchanged, and the geological conditions were from the same section. However, the RMS predicted values of TBM8 were generally slightly greater than the actual values, and the relative errors of the predictions were relatively large, with a maximum of 26.61% and a minimum of 2.76%. Different lithologies and equipment may cause fluctuations in RMS predictions. The average relative error of the predictions was 14.23%. Model validation and analysis results for TBM7 and TBM8 showed that the established prediction model was reliable.

**Figure 11:**
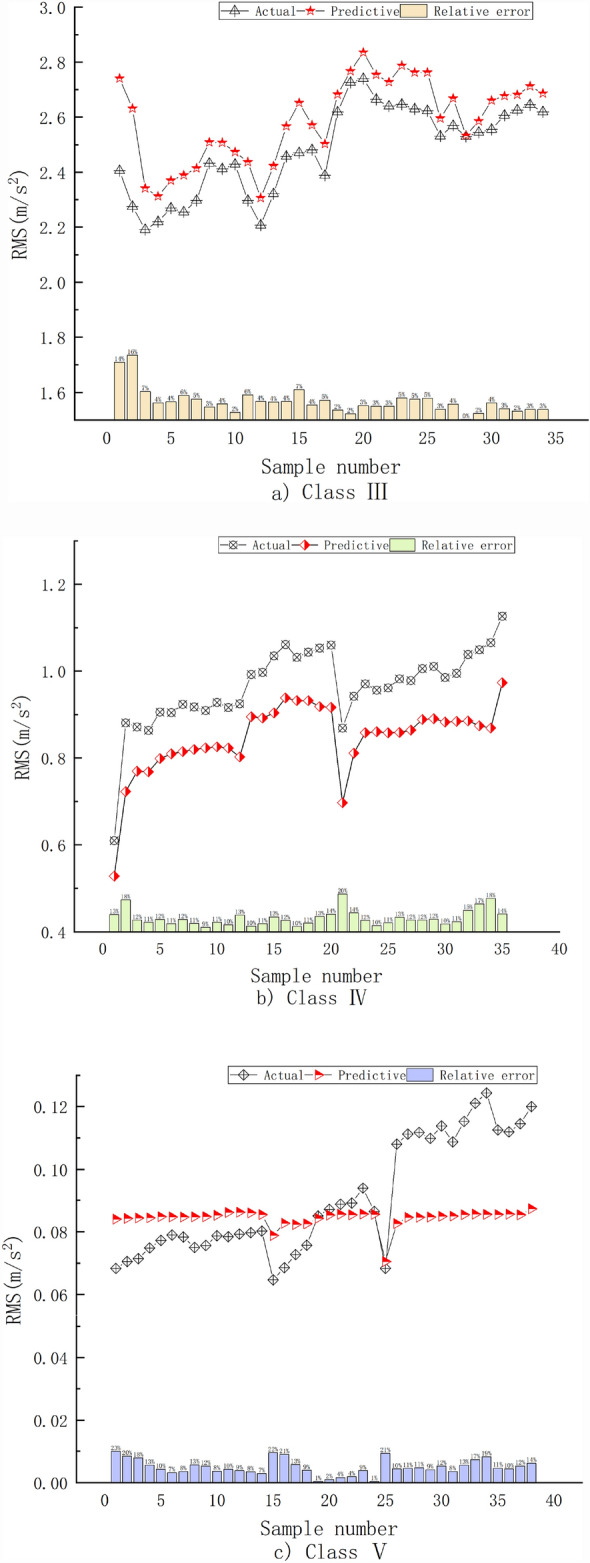
Comparison between the actual and predicted values for different surrounding rock types.

To further improve the prediction effect for different geological types and improve the universality of the prediction model, three tunnel sections of TBM7 were selected for collecting vibration signals during tunnelling, and the prediction model was validated and analysed. First, the pile numbers were 167 + 255.17–167 + 247.90, which included Class III surrounding rock with tuff and tuff breccia, and the rock mass strength was 70–80 MPa. Second, the pile numbers were 166 + 817.10–166 + 801.44, which included Class IV surrounding rock with tuffaceous sandstone lithology and a rock mass strength of 60–70 MPa. Third, the pile numbers were 168 + 782–168 + 760, which included Class V surrounding rock with carbonaceous shale lithology and a rock mass strength of 30–40 MPa. A comparison between the actual and predicted RMS values for the different surrounding rock types is shown in Fig. 11, and the RMS statistical analysis results are shown in Table 10.

**Table 10:** Statistics of the actual and predicted RMS values for the different surrounding rock types.

Class	Actual value			Predicted value			Relative error (%)		
	Max	Min	Mean	Max	Min	Mean	Max	Min	Mean
III	2.74	2.19	2.48	2.83	2.31	2.59	15.63	0.15	4.47
IV	1.13	0.61	0.96	0.97	0.53	0.85	19.75	9.46	12.36
V	0.124	0.06	0.09	0.09	0.07	0.08	23.04	0.90	11.30

As shown in Fig. 11 and Table 10, the variation trends of the RMS predicted values for different surrounding rock types were consistent with the variations in the actual values. For Class III surrounding rock, the maximum RE of the vibration RMS prediction was 15.63%, the average RE was 4.47%, and the prediction effect was the best. Thus, the geological conditions of the Class III surrounding rock and model were not significantly different. However, the prediction results for Class IV and Class V surrounding rocks were similar, with maximum REs of 19.75 and 23.04%, respectively, and average REs of 12.36 and 11.30%, respectively. The predicted RMS values for the Class IV surrounding rock were slightly lower than the actual values because the cracks in the Class IV surrounding rock were relatively developed, and large pieces of rock peeled off during rock breaking. This has a greater impact on the TBM, thus making the actual values slightly greater. For Class V surrounding rock, the variation range of the predicted RMS values was smaller than the actual value range. This was mainly because the rock was broken, and the rock would also be broken with lower tunnelling parameter values, resulting in a small fluctuation range of the tunnelling characteristic parameters. As a result, the predicted values of the RMS did not change significantly, whereas the actual values fluctuated during the acquisition process owing to the complex and changeable geological conditions. However, a comprehensive analysis of the RMS prediction errors of the vibrations for different surrounding rock types, lithologies, and equipment conditions showed that the established prediction model had good prediction accuracy and universality.

## Discussion

Considering the test conditions, influencing factors, application process, and practical requirements of the prediction model, the following four points should be stated:

Because it is rarely encountered in the actual construction process, Class I rocks were not considered in this study.

In this study, two TBMs with the same specifications from the same manufacturer were selected, and in situ excavation tests were conducted under the test conditions of typical tunnel sections. The number of samples collected under various geological conditions, such as different lithologies and rock mass strengths, is limited. In addition, the influences of the TBM equipment factors (such as the type and diameter) and the tunnel burial depth were not considered. Thus, the scope of application of the prediction model has certain limitations. However, in this study, a new method for obtaining the vibration magnitude of a TBM in real time was explored through the use of tunnelling characteristic parameters, and the influences of other factors need to be further studied.

In the practical application of the prediction model, several external environmental disturbances (such as anchor drilling rig construction, personnel walking, and external knocking), disc cutter wear, or other mechanical part failures will have a certain impact on the vibrations of the main beam of the TBM, resulting in a large deviation between the predicted results of the model and the actual vibration values. Thus, attention should be given to evaluating the prediction results in practical engineering site applications.

The relevant conclusions drawn in this paper have also been confirmed in the relevant literature^18,19^. However, this study examined the correlations between the tunnelling parameters and vibration characteristics of TBMs, determined the quantitative relationships between the tunnelling parameters and the RMS of the main beam vibrations of TBMs, and realized continuous real-time prediction of the main beam vibrations of TBMs. This effectively compensates for the lack of reliability in vibration-sensing monitoring.

## Conclusions

Using the China Xinjiang EH Project as an example, the influences of the excavation parameters and geological factors on the vibrations of the main beam of a TBM were comprehensively considered through theoretical analysis and in situ excavation tests. The correlations between the vibrations and tunnelling characteristic parameters were studied. A multiple-regression linear prediction model of the main beam vibration of the TBM was established, and the model was verified and analysed in field engineering. The following research results were obtained:

The main tunnelling parameters of the TBM, such as N, T, F, and P, significantly influence the vibrations of the main beam of the TBM. With an increase in the tunnelling parameters, the RMS also increased; that is, there were positive correlations between them.

The mapping relationships among the tunnelling parameters, characteristic parameters, and RMS were determined. There was an exponential relationship between P and the RMS, whereas F, T, FPI, and DPI had linear relationships with the RMS.

The RMS values were different for the different rock types. The RMS also increased with an increasing surrounding rock grade. The RMS was the largest for Class II surrounding rock, followed by those of Class III and IV surrounding rocks, and the RMS was the smallest for Class V surrounding rock. The RMS of the Class II surrounding rock was compared with that of the Class V surrounding rock, and the degree of variation in the RMS was 90%. Thus, there is a large range of values.

The ranges of the RMS correction coefficients for different surrounding rock types were calculated and determined based on the normalized vibration characteristics of the Class II surrounding rock.

The multiple linear regression prediction model was adapted to the different surrounding rocks using FPI and DPI as the characteristic parameters. The average relative error of the prediction was less than 12% after field engineering verification and analysis, which showed that the prediction model could accurately predict the vibrations of the main beam of the TBM.

In this paper, TBM excavation experiments were considered to be implemented in actual projects to study the relationship between the TBM vibration and excavation parameters under different surrounding rock conditions. Using excavation parameters such as the cutterhead speed (N), cutterhead torque (T), cutterhead thrust (F), and penetration (P) recorded by the TBM data acquisition system, the excavation characteristic parameters FPI and DPI are constructed. Furthermore, a relationship model relating the RMS vibration characteristics of the main beam of the TBM to the excavation characteristic parameters FPI and DPI is proposed. In this way, the prediction model proposed in this paper can perceive the magnitude of TBM vibrations in real time without the need to install traditional vibration sensing monitoring systems. Moreover, this approach effectively compensates for the shortage of monitoring interruptions caused by frequent failures of the sensor monitoring system owing to poor working conditions. This research explored a new method for TBM vibration monitoring and has important engineering significance.

## Data Availability

The data that support the findings of this study are available from the corresponding author, but restrictions apply to the availability of these data, which were used under licence for the current study and are not publicly available. However, the data are available from the authors upon reasonable request and with permission of the corresponding author.
